# *Bulbophyllum papuaense* (Orchidaceae), a new species from Indonesia

**DOI:** 10.3897/phytokeys.138.38714

**Published:** 2020-01-10

**Authors:** Dongliang Lin, Kailing Zhou, Arief Hidayat, Xiao-Hua Jin

**Affiliations:** 1 State Key Laboratory of Systematic and Evolutionary Botany, Institute of Botany, Chinese Academy of Sciences, Beijing 100093, China State Key Laboratory of Systematic and Evolutionary Botany, Institute of Botany, Chinese Academy of Sciences Beijing China; 2 Research Center for Biology, Indonesian Institute of Sciences, Cibinong, PO BOX 16911, Indonesia University of Chinese Academy of Sciences Beijing China; 3 Southeast Asia Biodiversity Research Institute, Chinese Academy of Sciences, Yezin, Nay Pyi Taw 05282, Myanmar Indonesian Institute of Sciences Cibilong Indonesia; 4 University of Chinese Academy of Sciences, Beijing, China Southeast Asia Biodiversity Research Institute, Chinese Academy of Sciences Yezin Myanmar

**Keywords:** *
Bulbophyllum
*, Indonesia, new species, Orchidaceae

## Abstract

A new species, *Bulbophyllum papuaense*, was described and illustrated from Indonesia. *Bulbophyllum papuaense* is similar to *Bulbophyllum tortuosum* and *B. muscohaerens* but differs from them by having rhizome and pseudobulbs covered with papillose scales, caudate and ciliate petals, linear and ciliate lip.

## Introduction

*Bulbophyllum* is among the largest genera of angiosperm, composed of more than 2200 species and widely distributed in tropical and subtropical regions throughout Africa, Asia, and the South Americas (Lindley 1830, Pearce and Cribb 2002, Seidenfaden 1979, 1992, Chen and Vermeulen 2009, Gravendeel and Vermeulen 2014, Chase et al. 2015, Govaerts et al. 2019, Vermeulen and O’Byrne 2011). *Bulbophyllum* is usually characterized by its creeping or pendent rhizomes with 1-internoded pseudobulbs, apex of pseudobulb with one or two non-sheathing leaves, inflorescences arising from nodes the rhizome, lateral sepals and column foot forming mentum, and waxy pollinia (Gravendeel and Vermeulen 2014).

Papua is the largest tropical island in the world and has a rich flora. It is estimated that there are 2869 orchid species in Papua (Ormerod 2017), most of which are endemic in Papua. For example, there are about 647 species in 36 sections of *Bulbophyllum* in New Guinea, out of which 590 species are endemic (Ormerod 2017). Most of these species are distributed in tropical montane forest (Ormerod 2017). During our fieldwork in montane forest in West Papua, Indonesia, in August 2016, a new species of *Bulbophyllum* was discovered and is described below.

## Taxonomy

### Bulbophyllum papuaense

Taxon classificationPlantaeAsparagalesOrchidaceae

X.H.Jin sp. nov.

821A9BEC-B953-5C46-8DD5-A7A8756850F5

urn:lsid:ipni.org:names:77204197-1

Figures 1 , 2 , 3

#### Type.

Indonesia. West Papua, Mokwan, Arfak Mountains, 1700–1900 m, August 16, 2016, Xiaohua Jin 17434 (holotype, BO; isotype, PE).

#### Diagnosis.

*Bulbophyllum papuaense* is close to *Bulbophyllum tortuosum* and *B. muscohaerens* but differs from them by having rhizome and pseudobulbs covered with papillose scales, caudate and ciliate petals, linear and ciliate lip which curve at the tip.

**Figure 1.**
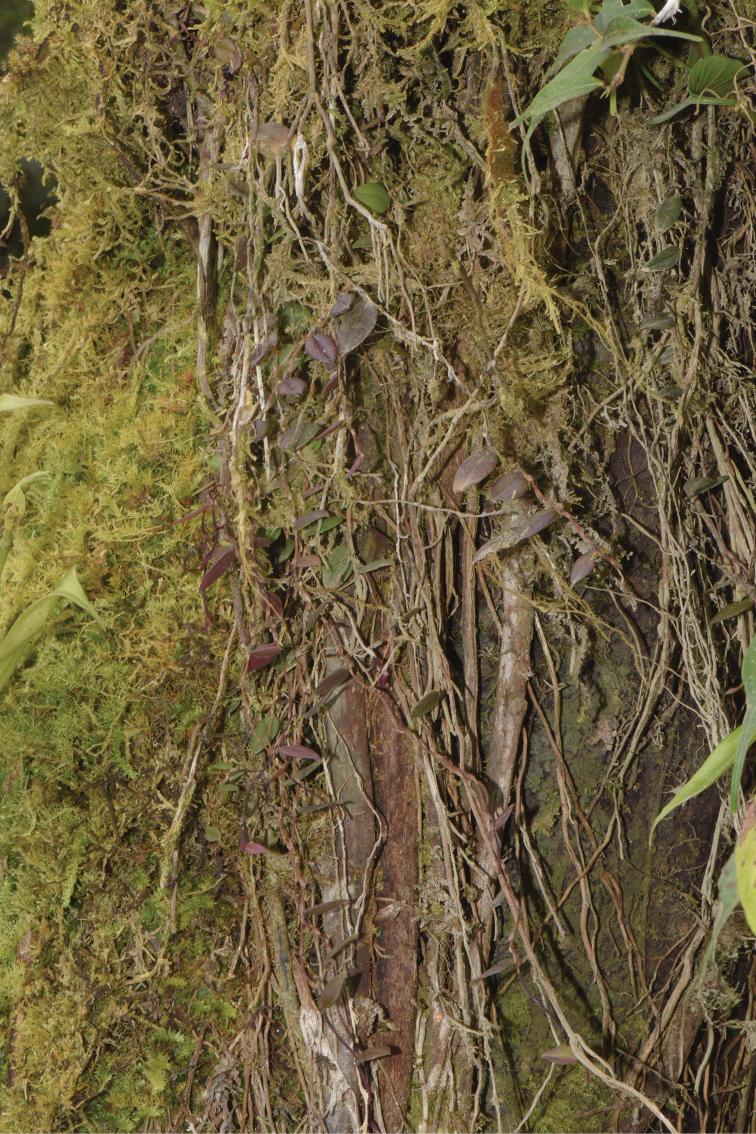
Habit of *Bulbophyllum papuaense*.

#### Description.

Epiphytic herb. Rhizome slender, creeping or pending with spreading roots, ca. 0.4 mm in diameter, brown, warty. Pseudobulbs elliptic, fleshy, ad-pressed to the stem, ca. 2.2 × 1.0 mm, with a long membranous sheath at base, usually 1-leaved. Leaf ca. 14 × 6.0 mm, elliptic to oval, entire, middle vein concave, apex mucronate, subsessile, young leaves green then turning reddish purple. Inflorescence solitary, usually 1-flowered, peduncle slender and short, ca. 1 mm long, base covered with long bract. Bracts tubular at base and caudate, 3–5 mm long. Flowers small, reddish purple, lateral sepals connate along their margins, together forming a somewhat boat-shaped structure, ca. 4.2 × 3.0 mm, margins entire, ciliolate in the proximal 1/2 from the base; median sepal oblong, apex attenuate to acuminate, ca. 5.5 × 2.0 mm, margin entire with obvious ciliate, 3-veined; petals much smaller than sepals, triangular and caudate, ca. 2.7 mm long, 1-veined, ciliate, apex contract to linear (caudate) and nearly 3 times as long as the basal part, margins with minutely white hairs; lip linear, recurved, dark reddish purple, ca. 2.6 mm long, with short white hairs, apex slightly widened with long and white hairs, a small triangular protuberance at base. Column white, including stelidia ca. 1.2 mm long, stelidia triangular, ca. 0.2 mm long, acute, with triangular and acute tooth along the upper margin; column foot 1 mm long; mentum cylindric, conspicuous, ca. 1 × 0.5 mm; pollinia 2.

**Figure 2.**
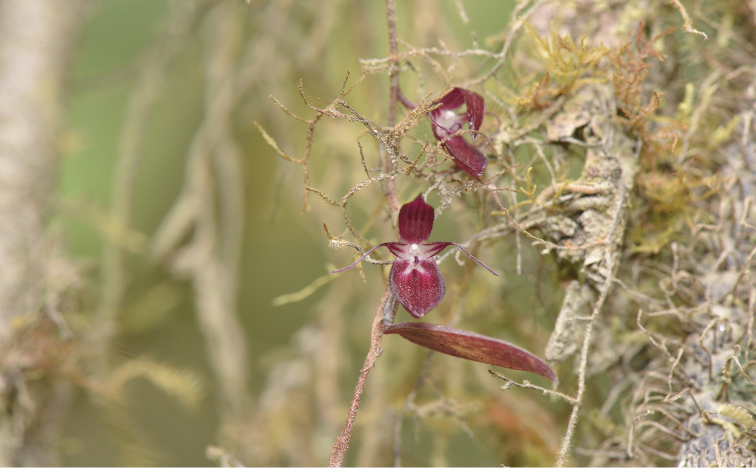
Close-up of flowers of *Bulbophyllum papuaense*.

#### Ecology.

*Bulbophyllum papuaense* was discovered in broad-leaved, evergreen montane forest in Mokwan, West Papua. *Bulbophyllum papuaense* is epiphytic on trunks or shrub in humid and shady areas in montane forest. Plants are tiny and grow usually with moss. Our observation indicated that it was in full bloom in August. *Bulbophyllum papuaense* is only known from the type locality.

#### Conservation status.

The tropical montane rain forest is well protected in Mokwan region. However, the rain forest is very difficult to reach due to poor transportation. Our examination in BO and Herbarium of Universitas Papua (Manokwari) did not find other collections of this species. Therefore, this new species is currently considered as DD.

#### Etymology.

The name derives from the Papua, where the new species was discovered.

#### Taxonomic notes.

*Bulbophyllum papuaense* belongs to sect. Oxysepala which is often characterized by 1-flowered inflorescence, lateral sepals connate, basal node of pedicel near at same level with the attachment of floral bract (Vermeulen et al. 2015). *Bulbophyllum papuaense* is a distinctive species in sect. Oxysepala and easily differs from its relatives by its morphological characters, such as the rhizome with warty scales, caudate and ciliate petals, linear lip recurve and with white hairs.

**Figure 3.**
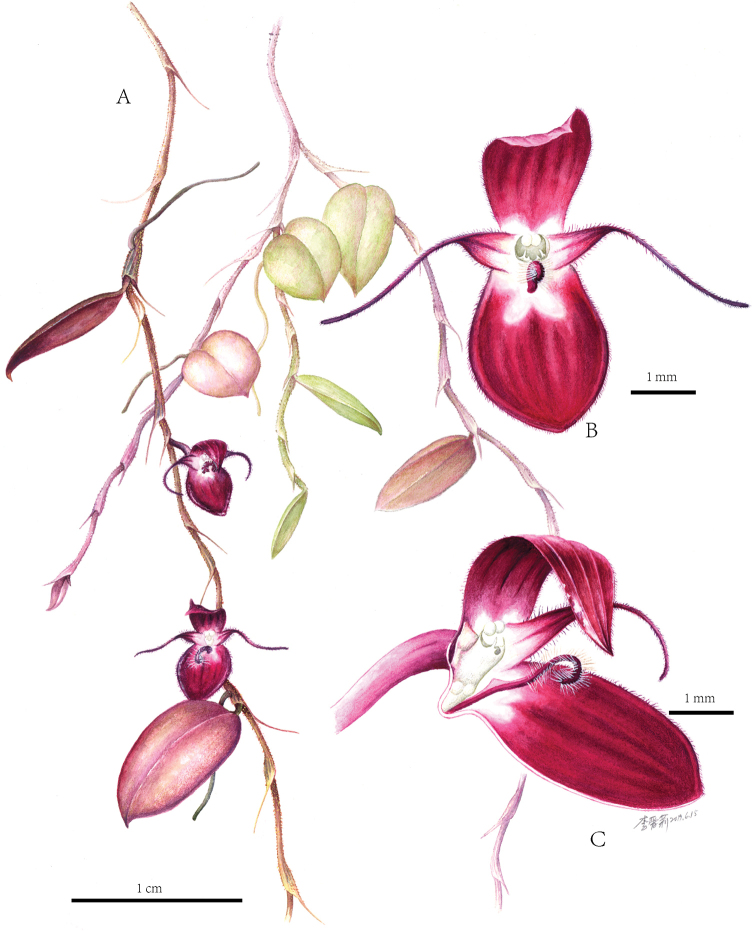
Color drawing of *Bulbophyllum papuaense***A** Plants **B** front view of flower **C** lateral view of flower.

### Key to *Bulbophyllum papuaense* and its alliance

**Table:** 

1	Lateral sepals forming a boated-shaped structure by the adherence of their lower margins	**2**
–	Lateral sepals free from each other adherent along their lower margins forming a boated-shaped structure	***B. leptoglossum***
2	Rhizome elongate, pseudobulbs well-spaced along the rhizome	**3**
–	Rhizome short, pseudobulbs clustered	***B. aberrans***
3	lip less than 0.5 mm wide, ration length/width about 10	**4**
–	lip more than 0.5 mm wide, ration length/width about 2	***B. tortuosum***
4	petals ciliate and caudate, lip ciliate along the margin and curve at tip	***B. papuaense***
–	petal (elliptic-) ovate, margin entire, lip ciliate at base	***B. muscohaerens***

## Supplementary Material

XML Treatment for Bulbophyllum papuaense
